# Supplementary material for: Molecular and Metabolic Insights into Anthocyanin Biosynthesis for Spot Formation on Lilium leichtlinii var. maximowiczii Flower Petals
Source: Int J Mol Sci. 2023 Jan 17;24(3):1844. doi: 10.3390/ijms24031844 (PMC9915866; doi:10.3390/ijms24031844)
Supplement: Supplementary file 1 [file ijms-24-01844-s001.zip › ijms-2129188-supplementary Figures.pdf]

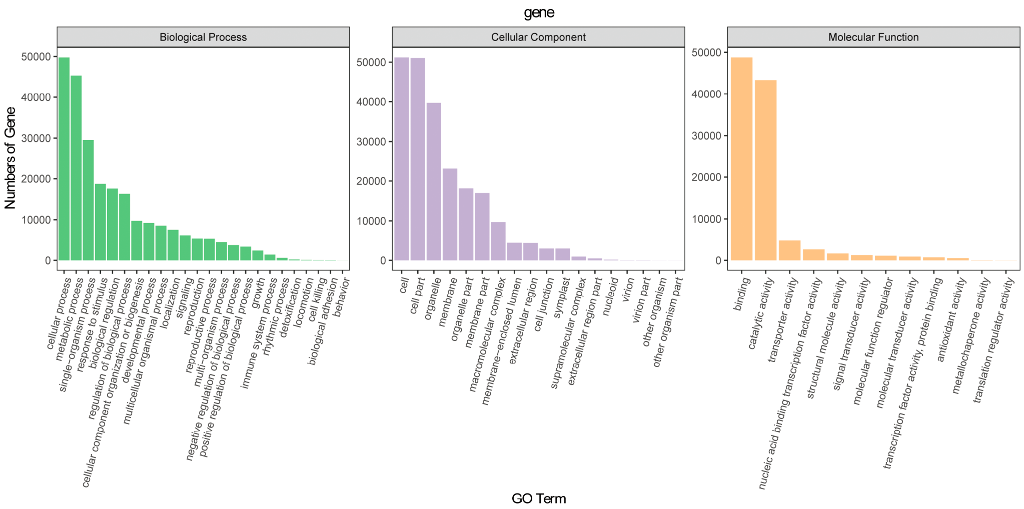

Figure S1: GO functional analysis and classification of L. leichtlinii var. maximowiczii transcriptome.

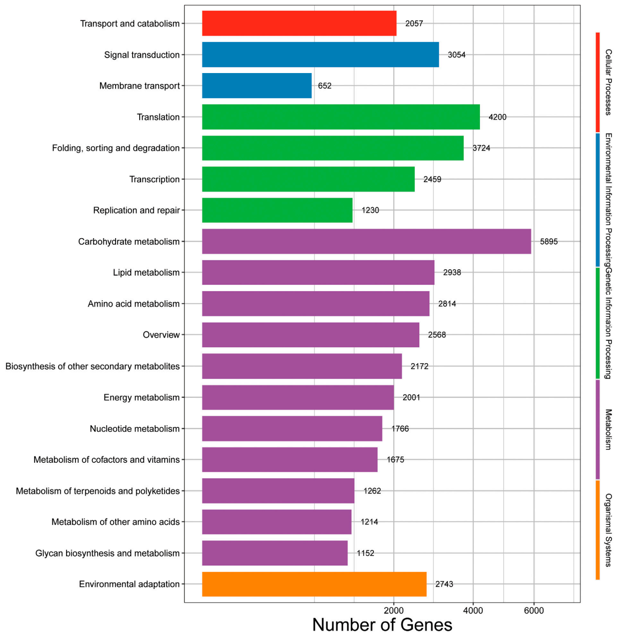

Figure S2: KEGG classification of L. leichtlinii var. maximowiczii transcriptome.
